# Antimicrobial Secretions of Toads (Anura, Bufonidae): Bioactive Extracts and Isolated Compounds against Human Pathogens

**DOI:** 10.3390/antibiotics9120843

**Published:** 2020-11-26

**Authors:** Candelario Rodriguez, Roberto Ibáñez, Louise A. Rollins-Smith, Marcelino Gutiérrez, Armando A. Durant-Archibold

**Affiliations:** 1 Centro de Biodiversidad y Descubrimiento de Drogas, Instituto de Investigaciones Científicas y Servicios de Alta Tecnología (INDICASAT AIP), Clayton, Panama City 0843-01103, Panama; Crodriguez@indicasat.org.pa; 2 Departamento de Bioquímica, Facultad de Ciencias Naturales, Exactas y Tecnología, Universidad de Panamá, Apartado 0824-03366, Panama; 3 Department of Biotechnology, Acharya Nagarjuna University, Nagarjuna Nagar, Guntur 522510, India; 4 Scientific Station COIBA, (COIBA AIP), Ciudad del Saber, Apartado 0816-02852, Panama; 5 Smithsonian Tropical Research Institute (STRI), Balboa 0843-03092, Panama; ibanezr@si.edu; 6 Departamento de Zoología, Facultad de Ciencias Naturales, Exactas y Tecnología, Universidad de Panamá, Apartado 0824-03366, Panama; 7 Department of Pathology, Microbiology, and Immunology, and Department of Pediatrics, Vanderbilt University School of Medicine, Nashville, TN 37232, USA; louise.rollins-smith@vanderbilt.edu

**Keywords:** alkaloids, amphibians, antimicrobial peptides, bufadienolides, Bufonidae, crude extract, parotoid gland, skin secretion, toad

## Abstract

Species of the family Bufonidae, better known as true toads, are widespread and produce bioactive substances in the secretions obtained from specialized skin macroglands. Some true toads have been employed as a folk remedy to treat infectious diseases caused by microbial pathogens. Recent publications based on in silico analysis highlighted the Bufonidae as promising sources of antimicrobial peptides. A review of the literature reveals that Bufonidae skin secretion extracts show inhibitory activity in vitro against clinical isolates of bacteria, resistant and standard strains of bacterial, and fungal and parasitic human pathogens. Secondary metabolites belonging to the classes of alkaloids, bufadienolides, and peptides with antimicrobial activity have been isolated from species of the genera *Bufo*, *Bufotes*, *Duttaphrynus,* and *Rhinella*. Additionally, some antimicrobial extracts and purified compounds display low cytotoxicity against mammal cells.

## 1. Introduction (True Toads, Antimicrobial Resistance of Human Pathogens against Drugs)

**Figure 1:**
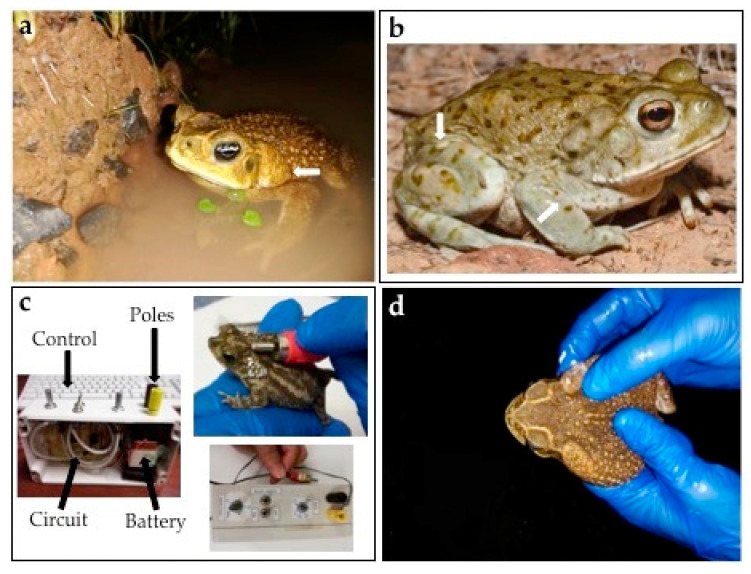
Species of true toads, with white arrows indicating skin macroglands. (**a**) Adult specimen of *Rhinella horribilis* (Wiegmann, 1833). (**b**) Adult toad of *Incilius alvarius* (Girard, 1859); (© 2014 Hunter McCall). (**c**) Mild electrical stimulation of an adult male of *Rhinella centralis* (Narvaes and Rodrigues, 2009) employing a transcutaneous amphibian stimulator [15]. (**d**) Stimulation of *Rhinella horribilis* by gentle compression of the parotoid glands.

Amphibians of the Bufonidae family are distributed worldwide, mainly in arid and glacial regions [1]. More than 600 species of the Bufonidae family have been classified into 50 genera, with the genus *Bufo* being the most abundant. [2]. Toads of the Bufonidae family are known as “true toads” because they possess specialized macroglands, identified as parotoids, behind the eyes [3]. Additionally, some true toads have macroglands on the limbs (Figure 1a,b).

The bufonid secretions contain small molecules such as alkaloids and steroids, as well as larger molecules such as peptides and proteins [4]. Skin gland secretions can be obtained by gentle manual compression, although other techniques have been successfully applied such as mild electrical discharges and chemical stimulation (Figure 1c,d). Amphibian stimulation should be carried out at intervals of 4 weeks in order to avoid harm to the animals [5]. Parotoid and skin glands of amphibians are involved in cutaneous respiration, reproduction, thermoregulation, and defense [6]. In vivo assays, via intraperitoneal injection in mice, revealed that parotoid gland secretions of *Rhinella marina* (Linnaeus, 1758) and *Rhaebo guttatus* (Schneider, 1799) contain metabolites that may act as antipredator agents through toxic and nociceptive effects [7]. True toads biosynthesize peptides and steroids that are able to inhibit the growth of amphibian pathogens. Three steroids, arenobufagin, gamma-bufotalin, and telocinobufagin, of the bufadienolide class were isolated from mucosal and skin gland secretions of the toad *Anaxyrus boreas* (Baird and Girard, 1852). All three bufadienolides showed activity against the lethal fungus of amphibians *Batrachochytrium dendrobatidis* (Bd). Furthermore, arenobufagin enhanced the growth of the anti-Bd bacterium *Janthinobacterium lividum* [8]. Two cathelicidin-derived peptides designated as BG-CATH(37) and BG-CATH(5-37) were identified from the DNA of the Asian toad *Bufo gargarizans* (Cantor, 1842). Both peptides were found to inhibit the aquatic bacteria *Vibrio splendidus*, *Streptococcus iniae,* and *Aeromorus hydrophila* that are usually found in the habitat of this toad [9].

Scientific research focused on the potential use of extracts and compounds isolated from true toads for medical treatment has increased during the last years [10]. Currently, clinical trials are underway to evaluate the anticancer activity of the aqueous extracts obtained from the dried skin of *Bufo gargarizans* [11]. Some evidence supports the use of bufonid secretions, found in true toads, in folk treatments for diseases caused by microbes. In the Colombian forest region of Cundinamarca, species of *Rhinella* toads are employed to treat erysipelas, which is an infection caused by the multidrug-resistant bacteria *Streptococcus pyogenes*. The treatment consists of rubbing the toad skin on the infected areas of the body in order to eradicate the infection [12]. Furthermore, *Rhinella marina* toads, from the Brazilian Amazon, have also been used for treatment of patients with erysipelas [13]. In the Northeastern region of Brazil, the skin and fat tissues from *Rhinella jimi* (Stevaux, 2002) are employed to treat asthma, cancer, and infections [14].

Due to the low efficacy of many antimicrobial drugs against many pathogenic microorganisms, the discovery of new antimicrobial compounds, such as peptide substances, has increasingly gained attention [16]. In this regard, amphibians are rich sources of antimicrobial peptides, and more than 100 of these chemical compounds have been discovered. These molecules confer to amphibians immunological, analgesic, antimicrobial, antiparasitic, hormonal regulation, mating, and wound healing properties for survival [17]. Recently, it was found by employing transcriptomic and peptidomic techniques that the deionized-water extract of the parotoid gland secretion from *B. gargarizans* contains a high amount of unique peptides, 23 of which showed defensive properties [18]. Antimicrobial peptides of the buforin, kassinin, temporin, peroniin, and rugosauperoilin families were identified from the DNA of the skin of the toad *Rhinella diptycha* (Cope, 1862). Caseinolytic activity was detected confirming the protease activity of toad skin secretions [19]. Furthermore, eight peptides with potential antimicrobial activities were predicted from the DNA sequence of H2A histones from the toads *Duttaphrynus melanostictus* (Schneider, 1799) and *Phrynoidis asper* (Gravenhorst, 1829). In silico analysis revealed that these peptides show a positively charged face, aggregation of bacterial cell membranes, and absence of cleavage sites for alpha-chymotrypsin, which suggests promising antimicrobial activity [20].

According to the World Health Organization (WHO) three of the top 10 causes of deaths worldwide are bacterial diarrhea, lower respiratory infections, and tuberculosis [21]. Fungal infections (mycosis) are opportunistic and affect mainly asthmatic, human immunodeficiency virus (HIV)-infected, and hospitalized patients. Current statistical data estimate 15,000 and 181,000 new cases worldwide per year of aspergillosis and cryptococosis, respectively [22]. Parasitic tropical diseases remain a challenge. There is a high incidence of leishmaniasis in 90 countries, with around eight million people at risk for American trypanosomiasis, 2000 positive cases between 2017 and 2018 of African trypanosomiasis, and about 1700 diagnosed cases each year of malaria in the United States, mostly in returning travelers [23]. Microbial illnesses that have been in the population but are increasing rapidly in prevalence and geographic localities are defined as reemerging infections [24]. The incidence of these diseases is mainly related to microbial adaptation, partly as a consequence of the inadequate and overuse of antimicrobial drugs by humans. Additionally, pathogenic bacteria, fungi, and parasites are able to develop strategies such as specific gene overexpression, incorporation of new virulence genes, and rapid genetic mutations to overcome biological pressures. These gene adaptions are then translated into proteins that confer microbial resistance [25].

Pathogenic microbes such as bacteria, fungi, and protozoan parasites may resist the bioactivity of drugs due to mechanisms related to membrane permeability, efflux systems, binding target, and drug inactivation [26]. The Gram-negative pathogen *Pseudomonas aeruginosa* changes membrane permeability against the antibiotic imipenem via deletion of D2 pores involved in drug transport across the plasma membrane [27]. The intracellular concentration of a drug decreases with the use of ATP-binding cassette (ABC) efflux pump systems in bacteria and represents a major cause of multidrug resistance [28]. Bacteria are able to develop modification to alter the drug-binding target interaction. Recently, it was reported that the *Mycobaterium tuberculosis* genome encodes 20 different cytochrome p450 (CYP) enzymes, which are involved in the biosynthesis of membrane steroids. Inhibition of CYP51B1 by rifampicin remains among the only few options for treatment of tuberculosis [29]. Gram-negative and Gram-positive pathogens produce enzymes that decrease the activity of antibiotics by modifying their chemical structure. Chloramphenicol-resistant strains of *Escherichia coli* and *Staphylococcus aureus* produce chloramphenicol acetyltransferase, an enzyme that catalyzes the acetylation of chloramphenicol into the inactive metabolite 1,2-di-acetyl-chloramphenicol [30]. Penicillin-resistant *S. aureus* strains deactivate β-lactam antibiotics via hydrolysis of the penam ring. β-Lactamases transform penicillins into penicilloic acids, which are not toxic for bacteria [31].

Fungal pathogens have been shown to produce efflux pumps at the plasma membrane for antimycotic azoles [32]. An ABC transporter was characterized in *Cryptococcus neoformans* and found to confer resistance against fluconazole [33]. Two genes that code for ABC transporters were identified in *Candida krusei*. Both genes display correlation with resistance against miconazole [34]. Alterations in the chemical composition at the membrane level in order to decrease susceptibility against antimycotics have been reported for human pathogenic fungi [32]. An amphotericin-resistant strain of *Aspergillus flavus* was produced experimentally from a susceptible wild-type strain. Chemical analysis of the cell membrane showed significant changes in chemical composition of carbohydrates, suggesting that acquired resistance is associated with modifications in membrane glycans [35]. Clinical isolates of *Candida albicans* that present mutations on the gen *erg3*, which encodes for ergosterol biosynthesis, were found to display resistance against amphotericin. The chemical analysis revealed highly decreased ergosterol content in comparison with polyene-sensitive strains [36].

*Trypansoma brucei,* the causative agent of African trypanosomiasis, exhibits cross-resistance against the drugs melarsoprol and pentamidine. This adaptation was revealed to be caused by the loss of the aquaporin-2 (AQP2) function at the cell membrane [37]. Genetic mutations on the *Plasmodium falciparum* chloroquine-resistant transporter (PFCRT) were detected in resistant clinical isolates [38]. Induced overexpression of the P-glycoprotein (LtrMDR1) from *Leishmania tropica* revealed that this protein pumps miltefosine out of the parasite cells, conferring resistance by decreasing intracellular drug concentrations [39]. The cellular disruption caused by metabolites produced by drug metabolism in parasites can be neutralized by the catalytic actions of cytosolic oxide-reductase enzymes. In this sense, the gene expression profile among sensitive and resistant strains of *Leishmania braziliensis* clinical isolates to antimony was evaluated. The results showed overexpression of the gene *TRYR*, which encodes the parasitic enzyme thrypanothione reductase [40].

Resistance mechanisms expressed by pathogenic microbes against drugs reveal the need for the development of more efficient treatments ideally focused on different targets not recognized by pathogens. In this sense, therapy based on bacteriophages, also known as phages, which are viruses that invade bacteria and disrupt the metabolism leading to bacterial lysis, has gained attention [41]. Another option is the research for new chemical entities able to inhibit growth and, hence, overcome the resistance that pathogenic microbes display. Bioactive compounds obtained from understudied natural sources such as toxins from amphibians, insects, marine invertebrates, spiders, and reptiles represent a promise for unknown chemical entities [42]. Former review articles described different bioactivities of compounds and extracts, isolated from the skin of amphibians, against several diseases [4,10]. Hence, our present work focused on a description and understanding, for the first time, of the importance of natural products isolated from true toads with potential for the development of antimicrobial drugs.

## 2. Methodology

This article reviews the literature of antimicrobial activity of molecules and extracts prepared from secretions of toads from the family Bufonidae. Selection of published information from the electronic databases PubMed. Gov, MDPI.com, SciFinder^®^, and ScienDirect.com, as well as Google Scholar, was carried out employing the key words “amphibians”, “antimicrobial secretions”, “animal venom”, “Bufonidae”, and “toads”. As result, this literature review covers papers from 1975 to 2020.

## 3. Antimicrobial Activities

Compounds and crude extracts prepared from skin secretions obtained from toads of the family Bufonidae display inhibitory activity against clinically isolated, drug resistant, and standard pathogenic strains of microbes (Table 1).

### 3.1. Antibacterial

**Figure 2:**
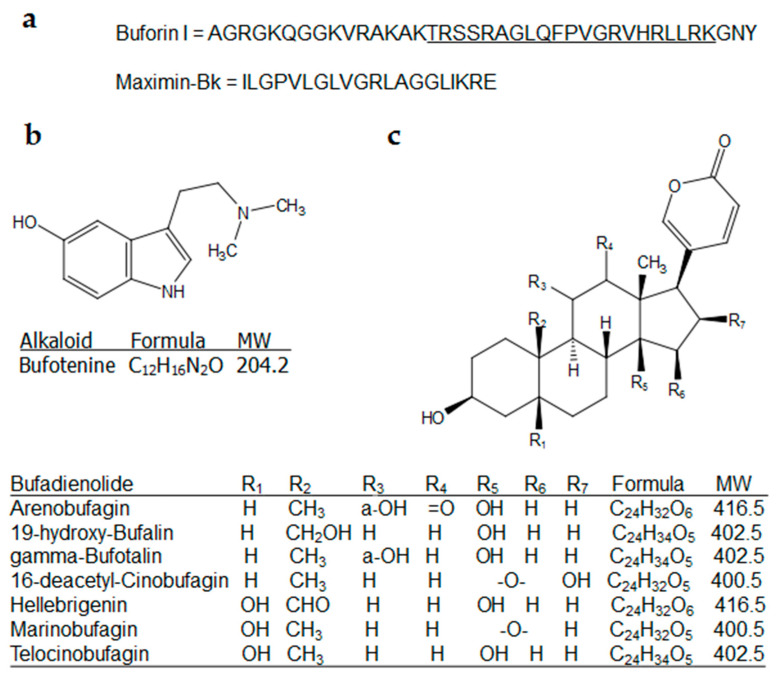
Chemical structures of antimicrobial molecules isolated from Bufonidae. (**a**) Primary structure of peptides; the underlined sequence in Buforin I represents the primary structure of buforin II. (**b**) Alkaloid. (**c**) Bufadienolides. MW: Molecular weight (g/mol).

The aqueous-soluble fraction of the parotoid gland secretion and the methanolic skin extract from the Sudan toad *Duttaphrynus melanostictus* have antibiotic potential against *Bacillus cereus*, *Escherichia coli*, *Klebsiella pneumoniae*, *Salmonella typhimurium*, *Staphylococcus aureus*, and *Staphylococcus epidermidis*; however, no activity was found against a methicillin-resistant strain of *S. aureus* [43,44]. The granular secretion from *Rhinella jimi* was extracted with 70% ethanol and evaluated against drug-resistant bacteria; however, no activity was observed. When tested in association with antibiotics of the aminoglycosides and β-lactam classes, the secretion was able to reduce the minimal inhibitory concentration (MIC) values of antibiotics against clinical isolates of *E. coli*, *Pseudomonas aeruginosa*, and *S. aureus* [45]. The dorsal skin secretion from *Phrynoidis asper* was extracted with deionized water and investigated for antibiotic potential. As a result, it was shown to have antibiotic properties with pronounced activity against the Gram-positive pathogens *S. aureus* and *Bacillus subtilis*. The protein profile of the antimicrobial skin secretion of *P. asper* was analyzed by tandem mass spectrometry (MS^2^). The bands from sodium dodecyl sulfate polyacrylamide gel electrophoresis (SDS-PAGE) wells were digested and showed fragments of proteins involved in different cellular processes, such as actins, cathepsins, histones, and synthases [46]. Buforin I, an antimicrobial peptide (AMP) with broad-spectrum activity, was isolated from the stomach of the toad *Bufo gargarizans* (Figure 2). Sequencing by Edman’s degradation revealed that Buforin I (MH^+^ = 4309 g/mol) and its endoproteinase Lys-C product, named Buforin II (MH^+^ = 2432 g/mol), are homologous to the N-terminal region of *Xenopus* histone H2A. Buforin II was found to have more potent bioactivity than Buforin I [47]. Labeling experiments showed that Buforin II is able to penetrate cell membranes of *E. coli* even at doses lower than its MIC value, as well as bind to RNA and DNA and, hence, disrupt cellular metabolism [48]. Structural–activity relationship analysis testing of synthesized analogues of Buforin II revealed that the proline hinge is essential for antimicrobial activity [49]. The skin secretion of the toad *Bufotes sitibundus* (Pallas, 1771) contains the peptides Buforin I, Maximin 1, Alyteserin-1a, and a novel +2 charged hydrophobic peptide named Maximin-Bk. This peptide has a molecular weight of 2012 g/mol (MH^+^) and showed antimicrobial activity with very low hemolytic activity against human erythrocytes even at 100 mg/mL [50]. Lectins with antibacterial activity LBP-1 (50 KDa) and LBP-2 (56 KDa) were isolated from skin of *Rhinella arenarum* (Hensel, 1867) via saccharide extraction. Microbial inhibition was determined to be bacteriostatic, and both proteins did not agglutinate human type O, A, and B erythrocytes [51]. Total aqueous crude secretion extracts were prepared by pooling skin (granular gland) and parotoid gland secretions followed by extraction with ultra-pure water. Total aqueous crude extracts obtained from *Bufo bufo* (Linnaeus, 1758), *Bufo verrucosissimus* (Pallas, 1814), and *B. sitibundus* displayed activity against *E. coli*, *S. aureus*, *Enterococcus faecalis*, *Enterococcus faecium*, *S. epidermidis*, and *S. thyphimurium* standard strains without hemolysis between 0.5 and 50 mg/mL [52]. A lysozyme (15 kDa) was isolated from the saline-soluble skin secretion extract of the Asian toad *B. gargarizans*. The protein was named Ba-lysozyme and exhibits potent bactericidal activity. The complete sequence of Ba-lysozyme was deduced by peptide mass fingerprinting and phylogenetic analysis. According to the occurrence in Ba-lysozyme of the amino-acid residues at positions glutamate-35 and aspartate-52, commonly found in other lysozymes and essential for lytic activity, the antibiotic mechanism of Ba-lysozyme is suggested to be via enzymatic degradation; however, the potent activity against both *S. aureus* (Gram+) and *E. coli* (Gram−) points to a different mechanism [53]. Solutions prepared from the parotoid gland secretion of *Rhinella icterica* (Spix, 1824) (no solvent reported) are able to inhibit the growth of *E. coli* and *S. aureus* in 15 and 30 min, respectively. This difference in antimicrobial action has been observed for some drugs, such as lincomycin and erythromycin, and may be related to differences in the structure of their cell walls [54]. Bufotenine, an indole alkaloid widespread among toads, shows antibacterial activity against *B. subtilis*, *E. coli*, *Proteus mirabilis,* and *S. aureus* with inhibition halos of 4, 6, 9, and 5 mm, respectively [55,56]. Two bufadienolides, known as marinobufagin and telocinobufagin, were isolated as major components from the chloroform/methanol (9:1)-soluble parotoid gland secretion extract of the toad *Rhinella rubescens* (Lutz, 1925). Both steroids presented antimicrobial properties with MIC values comparable to commercial antibiotics [57]. More recently, it was revealed that telocinobufagin was able to significantly decrease the bacterial burdens in spleen and enhance the Th1 immune response against the pathogen *S. typhimurium* in mice, as revealed by interferon gamma induction. Furthermore, after co-injection with formalin-inactivated *S. typhimirium*, telocinobufagin promoted the production of immunoglobulin G (IgG) and IgG2a antibodies, making it a promising adjuvant for antibiotic vaccines [58].

**Table 1:** Antimicrobial activity from true toads against human pathogenic bacteria, fungi, and parasites.

Toad Species ^a^	Sample (Solvent Used)	Effect	Bioactivity ^b,c,d^	Toxicity (μg/mL) ^d,e^	Reference
*Bufo bufo*	Skin gland + parotoid gland secretion (ultra-pure water)	Antibacterial	MIC (μg/mL): 250 (*S. thyphimirium*); 62.5 (*S. aureus*); 3.9 (*E. Faecalis*, *E. faecium*, *S. epidermidis*)	IC_50_: 0.35 (HEK-293)	[52]
		Antifungal	MIC (μg/mL): 250 (*C. albicans*)		
*Bufo gargarizans* (*Bufo andrewsi*)	Ba-lysozyme	Antibacterial	MIC (μM): 8 (*E. coli*); 1 (*S. aureus*)	*	[53]
*Bufo gargarizans*	Buforin-I	Antibacterial	MIC (μg/mL): 8 (*E. coli*, *Serratia* sp., *S. mutans*); 4 (*B. subtilis*, *P. putida*, *S. thyphimurium*, *S. aureus*, *S. pneumoniae*)	*	[47]
		Antifungal	MIC (μg/mL): 4 (*C. albicans*, *C. neoformans*)		
*Bufo verrucosissimus*	Skin gland + parotoid gland secretion (ultra-pure water)	Antibacterial	MIC (μg/mL): 62.5 (*S. aureus*); 3.9 (*E. faecalis*, *E. faecium*, *S. epidermidis*)	IC_50_: 0.99 (HEK-293)	[52]
		Antifungal	MIC (μg/mL): 125 (*C. albicans*)		
*Bufotes sitibundus* (*Bufo kavirensis*)	Maximin-Bk	Antibacterial	MIC (μg/mL): 20.78 (*L. mesenteroides*); 19.4 (*B. subtilis*); 18.5 (*B. cereus*); 16.3 (*S. aureus*); 10.3 (*P. aeruginosa*); 8.9 (*K. pneumoniae*); 8.1 (*E. coli*)	*	[50]
		Antifungal	MIC (μg/mL): 35.6 (*A. fumigates*); 32.1 (*C. albicans*); 28.6 (*A. niger*); 25.7 (*P. lilacinum*)		
*Bufotes sitibundus* (*Bufotes variabilis*)	Skin gland + parotoid gland secretion (ultra-pure water)	Antibacterial	MIC (μg/mL): 125 (*S. aureus*, *S. thyphimirium*); 7.8 (*S. epidermidis*); 3.9 (*E. faecalis*, *E. faecium*)	IC_50_: 1.46 (HEK-293)	[52]
		Antifungal	MIC (μg/mL): 125 (*C. albicans*)		
*Duttaphrynus melanostictus* (*Bufo melanostictus*)	Granular gland secretion (0.9% NaCl)	Antifungal	Halo zones (7.5% *w*/*v*): *C. albicans* (20.3 mm); *M. gypsum* (24.5 mm); *T. mentagrophytes* (24.7 mm)	*	[59]
	Parotoid gland secretion (distilled water)	Antibacterial	Halo zones: *E.coli* (7 mm); *S. typhimurium* (7 mm); *S. epidermidis* (8 mm); *K. pneumoniae* (9 mm); *B. cereus* (10 mm); *S. aureus* (11 mm)	*	[43]
	Skin (0.9% NaCl)	Antifungal	Halo zones: *P. notatum* (21 mm); *A. niger* (23 mm)	*	[60]
		Antibacterial	Halo zones: *E. coli* (19 mm); *K. pneumoniae* (24 mm); *S. aureus* (27 mm); *P. vulgaris* (33 mm)	*	[44]
	Skin gland secretion (distilled water)	Antibacterial	Halo zones: *K. pneumoniae* (19 mm); *E. coli* (25 mm); *P. vulgaris* (28 mm); *S. aureus* (30 mm)	*	[44]
		Antifungal	Halo zones: *P. notatum* (23 mm); *A. niger* (25 mm)	*	[60]
*Leptophryne cruentata*	Skin gland secretion (acetate buffer)	Antifungal	Halo zone: *T. mentagrophytes* (14.5 mm)	*	[61]
*Phrynoidis asper* (*Bufo asper*)	Skin secretion (deionized water)	Antibacterial	MIC (μg/mL): 100 (*E. coli*); 50 (*B. cereus*, *K. pneumoniae*, *P. aeruginosa*); 25 (*B. subtilis*); 12 (*S. aureus*)	*	[46]
*Rhaebo guttatus*	Skin gland secretion (CHCl_3_/MeOH)	Antiparasitic	IC_50_ (μg/mL): 0.05 (*P. falciparum*, ring)	LD_50_: 34.83 (BGM)	[62]
*Rhinella arenarum* (*Bufo arenarum*)	Venom (distilled water)	Antibacterial	MIC (μg/mL): >1250 (*A. baumannii*, *B. subtilis*); 1250 (*E. coli*, *K. pneumoniae*, *S. aureus*); 625 (*A. hydrophila*); 312.5 (*P. aeruginosa*)	*	[63]
		Antifungal	Inactive for *C. albicans* and *A. niger*		
	LBP-1	Antibacterial	Halo zones (25 μg): *E. coli* (16 mm); *E. faecalis* (12 mm); *P. morganii* (20 mm)	*	[51]
	LBP-2	Antibacterial	Halo zones (25 μg): *E. coli* (17.5 mm); *E. faecalis* (12.5 mm); *P. morganii* (19 mm)		
*Rhinella centralis*	19-Hydroxy-bufalin	Antiparasitic	IC_50_ (μg/mL): 7.81 (*T. cruzi*, tryp)	IC_50_: 71.58 (Vero)	[64]
*Rhinella icterica*	Parotoid gland secretion	Antibacterial	medium inhibition at 25 mg/mL for *E. coli*; *S. aureus*	*	[54]
*Rhinella jimi*	Parotoid gland secretion (EtOH)	Antibacterial	MIC (μg/mL): ≥2048 (*E. coli*; *P. aeruginosa*; *S. aureus*)	LD_50_: 365.94 (shrimp)	[45]
	Hellebrigenin	Antiparasitic	IC_50_ (μg/mL): 126.2 (*L. chagasi*, prom); 91.75 (*T. cruzi*, tryp)	IC_50_ > 200 (Macrophages)	[65]
	Telocinobufagin	Antiparasitic	IC_50_ (μg/mL): 61.2 (*L. chagasi*, prom)		
*Rhinella marina*	Skin gland secretion (crude)	Antibacterial	MIC (μg/mL): >25 (*E. coli*); 21 (*S. aureus*); 10.79 (*P. aeruginosa*)	IC_50_ > 100 (MRC5)	[66]
		Antiparasitic	IC_50_ (μg/mL): 14.82 (*L. braziliensis*, prom); 9.34 (*L. guyanensis*, prom); 2.43 (*P. falciparum*, ring)		
	Skin gland secretion (MeOH)	Antiparasitic	MIC (μg/mL): ≥100 (*L. braziliensis*, prom); 12.04 (*P. falciparum*, ring); 3.99 (*L. guyanensis*, prom)	*	[66]
	Skin gland secretion (CHCl_3_/MeOH)	Antiparasitic	IC_50_: 0.534 (*P. falciparium*, ring)	LD_50_ > 200 (BGM)	[62]
	16-Desacetil-cinobufagin	Antibacterial	MIC (μg/mL) < 3.12 (*E. coli*; *P. aeruginosa*; *S. aureus*)	IC_50_ > 100 (MRC5)	[66]
	Marinobufagin	Antibacterial	MIC (μg/mL) < 3.12 (*S. aureus*)	IC_50_ > 100 (MRC5)	[66]
	Telocinobufagin	Antiparasitic	IC_50_ (μg/mL): 1.28 (*P. falciparum*, ring)	IC_50_ > 200 (BGM)	[62]
*Rhinella rubescens* (*Bufo rubescens*)	Marinobufagin	Antibacterial	MIC (μg/mL): 128 (*S. aureus*); 16 (*E. coli*)	*	[57]
	Telocinobufagin	Antibacterial	MIC (μg/mL): 128 (*S. aureus*); 64 (*E. coli*)		
*Sclerophrys pantherina* (*Amietophrynus pantherinus*)	Skin gland secretion (phosphate buffer)	Antifungal	MIC (mg/mL): 0.39 (*F. verticillioides*); 0.04 (*C. albicans*); 0.02 (*A. flavus*)	*	[67]

^a^ Current names, scientific names in the original publications are in parentheses. ^b^ Abbreviations: Gram—(*A. baumannii*—*Acinetobacter baumannii*; *A. hydrophila*—*Aeromonas hydrophila*; *E. coli*—*Escherichia coli*; *K. Pneumoniae*—*Klebsiella pneumoniae*; *P. aeruginosa*—*Pseudomonas aeruginosa*; *P. vulgaris*—*Proteus vulgaris*; *P. morganii*—*Proteus morganii*; *S. thyphimurium*—*Salmonella thyphimurium*); Gram+ (*B. cereus*—*Bacillus cereus*; *B. subtilis*—*Bacillus subtilis*; *E. faecalis*—*Enterococcus faecalis*; *E. faecium*—*Enterococcus faecium*; *L. mesenteroides*—*Leuconostoc mesenteroides*; *P. putida*—*Pseudomonas putida*; *S. aureus*—*Staphylococcus aureus*; *S. epidermidis*—*Staphylococcus epidermidis*; *S. mutans*—*Streptococcus mutans*; *S. pneumoniae*—*Streptococcus pneumoniae*); fungi (*A. flavus*—*Aspergillus flavus*; *A. fumigatus*—*Aspergillus fumigatus*; *A. niger*—*Aspergillus niger*; *C. albicans*—*Candida albicans*; *C. neoformans*—*Cryptococcus neoformans*; *F. verticillioides*—*Fusarium verticillioides*; *M. gypsum*—*Microsporum gypsum*; *P. lilacinum*—*Penicillium lilacinum*; *P. notatum*—*Penicillium notatum*; *T. mentagrophytes*—*Trichophyton mentagrophytes*); protozoas (*L. braziliensis*—*Leishmania braziliensis*; *L. chagasi*—*Leishmania chagasi*; *L. guyanensis*—*Leishmania guyanensis*; *P. falciparum*—*Plasmodium falciparum*; *T. cruzi*—*Trypanosoma cruzi*). Drug-resistant or clinically isolated strains are in bold. ^c^ Parasitic stages: prom: promastigotes, tryp: trypomastigotes, ring: ring stage; ^d^ MIC: minimal inhibitory concentration; IC_50_: half maximal inhibitory concentration; LD_50_: median lethal dose. ^e^ Toxicity model description: BGM, kidney glomerular cells; HEK-293, noncancerous kidney cells; macrophages, BALB/C mice macrophages; MRC5, lung human normal fibroblasts; shrimp, Artemia salina larvae; Vero, epithelial kidney monkey cells. * Not evaluated.

### 3.2. Antifungal

Screenings of crude skin secretions from bufonids have become a recent field of research for novel sources of antimycotics. The aqueous-soluble parotoid gland secretions from the toads *B. bufo*, *B. verrucosissimus*, and *B. variabilis* inhibit the growth of *Candida albicans* ATCC 10239. Additionally, extracts from these toads were evaluated for hemolytic activity on red blood cells from healthy rabbits, and no toxicity was detected [52]. The methanol-soluble skin extract and the water-soluble parotoid gland secretions from the toad *D. melanostictus* were found to be active with a similar inhibition halo against *Aspergillus niger* and *Penicillium notatum* [60]. Micrographs of the fungal morphology after treatment with the *D. melanostictus* secretions prepared by extraction with physiological saline revealed disrupted integrity of the cell wall, which was observed as pore formation and shrinkage of the membrane [40]. The human pathogen *Trichophyton mentagrophytes* is inhibited by the saline phosphate buffer-soluble extract of the skin secretion from the toad *Leptophryne cruentata* (Tschudi, 1838). According to gas chromatography (GC) analysis, *L. cruentata* skin secretions contain amines, fatty acids, and steroids; however, no indole alkaloids were detected [61]. Toads of the family Bufonidae biosynthesize indole alkaloids that may represent as much as 15% of the dried weight of skin gland secretions [68]. The antifungal effects of amphibian alkaloids, such as samandarines and indoles, were evaluated on the nonpathogenic fungus *Saccharomyces cerevisiae*. The results showed that the alkaloids induced disturbances in the cytoplasm and plasma membrane, as observed by the appearance of vacuoles and translucent bodies in the cytoplasm [56]. The skin secretion from *Rhinella jimi* (Stevaux, 2002) was purified by Soxhlet extraction. The fixed oil obtained was evaluated in combination with some antifungal drugs. The oil extract was able to increase the potency (MIC) of amphotericin-B from 512 to 64 µg/mL against *Candida krusei* ATCC 6258. The chemical analysis by GC revealed that the fixed oil of *R. jimi* is composed mainly of the methyl esters of linoleic, oleic, palmitic, and stearic fatty acids [69]. Recently, the antifungal properties of some methyl esters against human pathogenic fungi were revealed [70]. The skin secretion of the South African toad *Sclerophrys pantherina* (Smith, 1828) was extracted with saline buffer phosphate and was found to be bioactive against the pathogens *Aspergillus flavus*, *C. albicans*, and *Fusarium verticillioides*. A temporal analysis of the MIC values suggested that the toad secretion inhibited fungal growth by killing the fungus, as revealed by stable inhibition for 120 h [67]. Two peptides with antifungal properties have been isolated from bufonid toads. Buforin I from *B. gargarizans* was equipotent against standard strains of *C. albicans*, *Cryptococcus neoformans*, and *S. cerevisae* [47]. Maximin-Bk from *B. sitibundus* was able to inhibit *Aspergillus fumigatus*, *A. niger*, *C. albicans*, and *Penicillium lilacinum* at nanomolar levels. On the basis of the observed low MIC values, researchers suggested that the inhibitory activity of Maximin-Bk is carried out via fungicidal effects [50].

### 3.3. Antiprotozoal

The antimicrobial potential of bufadienolides from toads was initially ignored and believed not to possess significant inhibitory activities against human pathogens [71]. Hellebrigenin and telocinobufagin were extracted from the skin secretion of the toad *R. jimi* via bioguided isolation. Both bufadienolides inhibit the growth of *Leishmania chagasi*, but only hellebrigenin was active against *Trypanosoma cruzi*. According to electron microscopy analysis, antileishmanial activity of telocinobufagin is mediated by damage to mitochondria and plasma membranes of the parasites [65]. The parotoid gland secretion of the Panamanian toad *Rhinella centralis* contains 19-hydroxy-bufalin as a major component. In vitro bioassays revealed that 19-hydroxy-bufalin exerted growth inhibition of *Trypanosoma cruzi* with significant selectivity, as its cytotoxicity was limited against normal kidney Vero cells [64]. The chloroform/methanol extract prepared from the parotoid gland secretion of the toads *Rhinella marina* (Linnaeus, 1758) and *Rhaebo guttatus* (Schneider, 1799), as well as telocinobufagin, displays antimalarial activity against the cloroquine-resistant strain W2 of *Plasmodium falciparum*. Remarkably, toad poisons and telocinobufagin presented low cytotoxicity against human cells and high selectivity for parasites [62]. Recently, the crude (no solvent reported) and methanolic extracts from the *R. marina* parotoid secretion showed antileishmanial and antiplasmodial activity. In general, both extracts displayed antiparasitic activity, although the methanolic extracts showed less inhibition against *Leishmania braziliensis* promastigotes. Biological evaluations revealed that *R. marina* crude extract did not induce DNA damage or mutagenesis [66].

## 4. Concluding Remarks

Bioactive molecules produced by bufonids in skin gland secretions as a defense mechanism against pathogens and predators have gained interest for drug discovery and development. Traditional uses of true toads as medicine for infections caused by microbes highlight the potential of bufonids as source of antimicrobial drugs. Bioguided isolation has allowed purification of alkaloids, peptides, and steroids with activity against human pathogens. Additionally, recent publications using omics technologies of gland and skin secretions have demonstrated that bufonids produce peptides with unknown structures, as well as peptides with known antimicrobial activity. Microbial resistance against drugs continues to be an important factor in the reemergence of infectious diseases. Crude extracts and isolated metabolites such as alkaloids, peptides, and steroids from Bufonidae display potent activity against clinical isolates, resistant and standard strains of bacteria, fungi, and protozoan parasites. An elucidation of biochemical mechanisms involved in the reported microbial inhibition by true toad secondary metabolites is needed. Bufonidae skin gland secretions represent a valuable source of antimicrobial agents with potential for the development of novel therapeutic drugs in studies against pathogens.
